# Laterality of hand-arm vibration exposure during grinding operations: a field study in industrial settings

**DOI:** 10.47626/1679-4435-2026-1593

**Published:** 2026-08-27

**Authors:** Marcos Jorge Gama Nunes, Osvaldo Luiz Gonçalves Quelhas, Maria de Lurdes Costa Domingos, Igor Macedo de Lima, Luiz Carlos de Miranda-Junior

**Affiliations:** 1 Universidade Federal Fluminense (UFF), Departamento de Engenharia, Niterói, RJ, Brazil; 2 Universidade Estadual de Campinas (UNICAMP), Faculdade de Tecnologia, Campinas, SP, Brazil

**Keywords:** hand-arm vibration, noise, occupational exposure.

## Abstract

**Introduction:**

Occupational exposure to hand-arm vibration generated by the use of powered hand tools is a major risk factor for workers’ health, particularly when combined with high levels of occupational noise. Among these tools, angle grinders used by welders are recognized as important sources of both physical hazards.

**Objectives:**

To investigate the laterality of hand-arm vibration exposure among workers using 7-inch and 9-inch electric angle grinders by comparing vibration exposure between the control hand positioned on the rear handle and the supporting hand positioned on the front handle as well as to assess the intensity of occupational noise generated by these tools.

**Methods:**

A retrospective observational study was conducted involving six welders from three companies representing different industrial sectors under real-world working conditions. Vibration and noise measurements were performed in accordance with the procedures established by Brazilian Occupational Hygiene Standard 10 and Brazilian Occupational Hygiene Standard 01, using the normalized resultant acceleration and A-weighted sound pressure levels expressed in dB(A).

**Results:**

Hand-arm vibration exposure showed an asymmetric pattern, with higher vibration levels consistently recorded at the rear handle (control hand), regardless of tool size or grinding disc type. In all samples, vibration levels exceeded the action value and, in some cases, surpassed the occupational exposure limit established by Brazilian legislation. Noise levels also exceeded the action value, with no evidence of lateral predominance.

**Conclusions:**

These findings highlight the importance of measuring vibration exposure in both hands and reveal gaps in the current regulatory framework regarding objective criteria for the simultaneous assessment of hand-arm vibration exposure.

## INTRODUCTION

Powered hand tools have become indispensable across a wide range of industrial sectors because they reduce both the time required to perform tasks and the physical effort involved in manufacturing and maintenance operations [^1^]. Among these tools, angle grinders used by welders for metal grinding are recognized as major sources of occupational exposure to hand-arm vibration (HAV) and noise [^1^,^2^]. Operating these tools requires sustained grip force on the trigger and handles, resulting in the direct transmission of vibration to the upper limbs throughout tool operation [^1^,^2^].

Prolonged exposure to HAV has been associated with a wide range of adverse health outcomes, including musculoskeletal disorders, neurosensory impairments, vascular diseases, and hearing loss, particularly under conditions of concurrent noise exposure [^1^,^3^,^4^]. Recent evidence indicates that workers simultaneously exposed to vibration and noise are at greater risk of developing hearing loss than those exposed to noise alone, suggesting a synergistic interaction between these physical agents [^5^,^6^].

In Brazil, Regulatory Standard (NR) 15 establishes guidelines for assessing and controlling occupational exposure to HAV, specifying an action value of 2.5 m/s^2^ and an occupational exposure limit of 5.0 m/s^2^ for the normalized exposure acceleration (aren in Portuguese) [^7^]. Complementing this regulation, Brazilian Occupational Hygiene Standard (NHO) 10 provides procedures for assessing HAV exposure and recommends that, when substantial differences are identified between the aren values affecting each hand, measurements should be performed on the hand subjected to the higher exposure level [^8^]. However, the standard does not define objective criteria for what constitutes a “substantial difference” between hands, nor does it require simultaneous measurements on both handles of tools operated with a control hand and a supporting hand. This regulatory gap creates uncertainty regarding the practical implementation of these technical guidelines in occupational exposure assessment [^8^,^9^].

Studies involving brush cutters, leaf blowers, pole pruners, and chainsaws have consistently shown that the handle operated by the right hand generally exhibits higher aren values than the supporting handle, demonstrating an asymmetric distribution of vibration exposure between the upper limbs [^1^,^5^,^10^].

Despite advances in characterizing occupational exposure associated with tools used in landscaping, construction, and forestry, field studies investigating the laterality of HAV exposure together with occupational noise levels during electric angle grinder operations performed by welders under real-world working conditions remain scarce [^2^,^11^,^12^].

Consequently, a significant gap persists in the scientific literature owing to the lack of objective criteria for determining which hand is subjected to greater vibration exposure, thereby hindering the standardization of decision-making in occupational hygiene practice. Accordingly, the present study aimed to investigate the laterality of HAV exposure among welders using 7-inch and 9-inch electric angle grinders under real-world working conditions by comparing exposure levels between the control and supporting hands and examining their relationship with occupational noise exposure.

## METHODS

The present study was designed as a retrospective observational investigation based on the secondary analysis of technical data extracted from occupational noise and HAV assessment reports. Data were originally collected as part of routine occupational hygiene evaluations rather than for research purposes. Subsequently, data were systematically compiled and comparatively analyzed in light of the scientific literature to investigate the laterality of HAV exposure and the associated levels of occupational noise while preserving worker anonymity.

The retrospective analysis included data from three companies and focused on welders exposed to HAV during the operation of large electric angle grinders equipped with 7-inch and 9-inch grinding discs (Chart 1). A purposive convenience sample was used based on the availability of technical occupational hygiene reports, an approach inherent to retrospective observational studies using secondary data. Consequently, no attempt was made to ensure statistical representativeness of the findings [^13^].

**Chart 1:** Field sample data

Company	Sample	Date of data collection	Equipment and model	Activity performed	aren (m/s^2^) Point 1 - Front handle (supporting hand)	aren (m/s^2^) Point 2 - Rear handle (control hand)	Noise (NEL) [dB(A)] Point (1)	Noise (NEL) [dB(A)] Point (2)
Logistics (Workshop)	Welder 1 (right-handed): ; weight: 73 kg	07/31/2025	DeWALT^®^ DWE 4577 N-B2 angle grinder, 2,700 W, 7-inch	Grinding of an irregularly shaped metal workpiece secured to a workbench (grinding disc)	3.70	3.80	101.0	92.3
Logistics (Workshop)	Welder 2 (left-handed): ; weight: 75 kg	07/31/2025	DeWALT^®^ DWE 4577 N-B2 angle grinder, 2,700 W, 7-inch	Grinding of a regularly shaped metal workpiece positioned horizontally and secured to a workbench (grinding disc)	4.40	4.70	99.4	99.5
Logistics (Workshop)	Welder 3 (right-handed): ; weight: 76 kg	06/08/2025	DeWALT^®^ DWE 4577 N-B2 angle grinder, 2,700 W, 7-inch	Grinding of a regularly shaped metal workpiece using different tool application angles (grinding disc)	2.80	3.90	99.8	100.3
Engineering Services	Welder 4 (right-handed): ; weight: 83 kg	09/30/2025	Bosch^®^ GWS 30-230 PB angle grinder, 2,800 W, 9-inch	Grinding of a regularly shaped metal workpiece using multiple tool application angles (Norton^®^ BDA 640 grinding disc)	5.90	10.50	90.0	91.3
Engineering Services	Welder 5 (right-handed): ; weight: 115 kg	10/01/2025	Bosch^®^ GWS 30-230 PB angle grinder, 2,800 W, 9-inch	Grinding of a regularly shaped metal workpiece using multiple tool application angles (Norton^®^ BDA 640 grinding disc)	4.50	6.70	96.3	93.7
Automotive - Maintenance - Welding	Welder 6 (right-handed): ; weight: 85 kg	10/30/2025	Makita^®^ M0920 angle grinder, 2,200 W, 7-inch	Surface finishing of semi-finished metal components using a Norton^®^ flap disc	5.20	9.10	84.4	88.3

Source: Prepared by the authors (2026).

aren = normalized exposure acceleration; HAV = hand-arm vibration; NEL = Normalized Exposure Level; NR = Regulatory Standard.

The action value for HAV exposure is 2.5 m/s^2^ (aren), and the tolerance limit is 5.0 m/s^2^ (aren). For occupational noise, the tolerance limit is 85 dB(A) for an 8-hour reference workday (NEL).

The sample comprised three welders from a logistics company, two welders from an engineering services company, and one welder from an automotive company, all belonging to the same occupational category. Variables related to exposure laterality, simultaneous use of both hands, working posture, exposure duration, and tool type were analyzed. For comparative purposes, a 4-hour daily exposure period was considered, with values expressed as the resultant acceleration of normalized exposure (aren) for the 8-hour reference workday, in accordance with NHO 10. All technical measurements had been conducted before the development of the present study as part of routine occupational hygiene assessments, with no influence of the research process on work activities.

Data were collected under real-world working conditions during grinding operations performed by the welders, following the procedures and criteria established by NHO 01 and NHO 10 for the assessment of occupational noise and HAV exposure, respectively.

The following equipment was used:

- Chrompack noise dosimeters compliant with ANSI S1.25, IEC 60804, IEC 61672, IEC 60651, IEC 61252, IEC 61260, and NHO 01, configured to determine the time-weighted average exposure level.
- An occupational vibration meter compliant with ISO 8041, ISO 2631, ISO 5349, NHO 09, NHO 10, and European Directive 2002/44/EC, configured to determine the resultant acceleration and the normalized exposure acceleration (aren).

**Figure 1:**
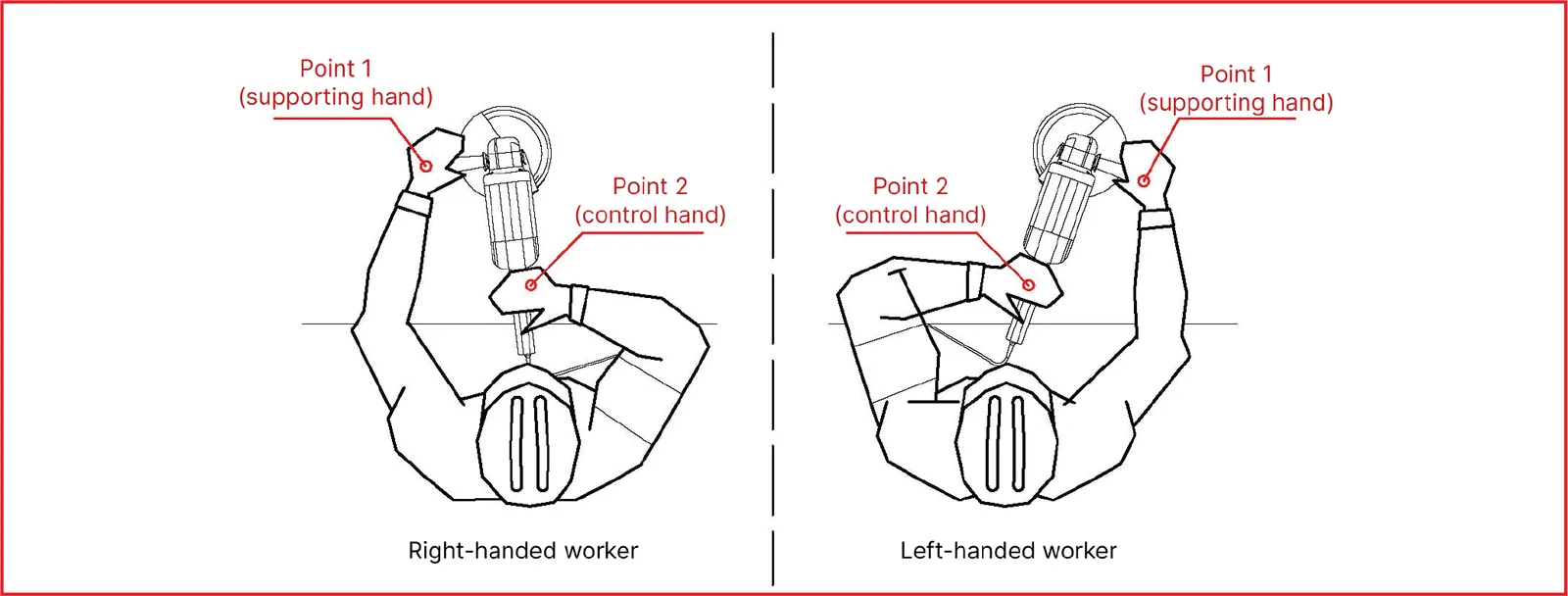
Positioning of hand-arm vibration measurement points at the front handle (point 1) and rear handle (point 2) in rightand left-handed workers.

The measurement of occupational noise and HAV followed a methodological approach similar to that reported in international studies, adapted to the operational conditions of the welders evaluated [^2^]. Six welders with different body statures were assessed while operating the angle grinder models routinely used in their work activities. An accelerometer mounted on a positioning adapter was first attached to the right hand and subsequently to the left hand during normal tool operation, allowing triaxial acceleration to be recorded for each upper limb. Simultaneously, two noise dosimeters measured sound pressure levels on both sides of the workers’ hearing zone, enabling the assessment of the noise generated during tool operation as well as any lateral differences in noise exposure (Figure 1).

Measurements were obtained through direct observation of grinding operations, encompassing the different stages of the work process and the postures adopted by the workers while performing their tasks. Potential factors that might influence exposure patterns (including the specific grinding disc used in each operation, the use of grinders powered by alternative energy sources, workers’ level of training, the presence of laterality-related hearing loss, and the use of anti-vibration gloves) were not investigated.

The vibration exposure obtained were subsequently compared with the action value and tolerance limit established by Annex 8 of NR 15 for occupational exposure to HAV. Noise exposure levels, normalized to a reference 8-hour workday, were compared to the tolerance limit of 85 dB(A), in accordance with the criteria of NHO 01 and Annex 1 of NR 15.

## RESULTS

Chart 1 presents the demographic and occupational characteristics of the study participants, together with the results of the HAV and noise assessments. All participants were male welders with previous experience in the occupation and were not apprentices. Mean height was 1.74 m (range: 1.60-1.85 m), and mean body weight was 84.5 kg (range: 73-115 kg).

All six scheduled monitoring days were successfully completed, resulting in a 100% complete data acquisition rate. During the data collection phase, one sample was excluded because a worker used a 4-inch angle grinder that differed from the model specified in the study protocol. Owing to this change in the exposure conditions, the corresponding assessment was repeated to ensure procedural standardization and comparability across samples.

The evaluated activities comprised typical grinding operations performed under different working conditions, including variations in operator posture, workpiece geometry, and tool application angle, predominantly using grinding discs. In one assessment, a Norton flap disc was used exclusively for the surface finishing of semi-finished metal components.

Across all samples, aren values ranged from 2.80 to 5.90 m/s^2^ at the front handle (point 1), with a mean of 4.42 m/s^2^, and from 3.80 to 10.50 m/s^2^ at the rear handle (point 2), with a mean of 6.45 m/s^2^. In all evaluated conditions, the highest vibration levels were recorded at the rear handle.

Occupational noise levels ranged from 84.4 to 101.0 dB(A) at point 1, with a mean of 95.2 dB(A), and from 88.3 to 100.3 dB(A) at point 2, with a mean of 94.0 dB(A). The mean values observed at both points exceeded the tolerance limit for an 8-hour reference workday. In contrast to vibration exposure, no consistent predominance of noise exposure was observed between the two measurement points.

In the Engineering Services sector, Welder 4 (1.80 m tall; 83 kg) exhibited the highest vibration exposure levels among all analyzed samples. While operating a 9-inch Bosch^®^ GWS 30-230 PB angle grinder, the highest-powered tool used in this study, equipped with a Norton BDA 640 grinding disc, and performing grinding operations on a regularly shaped metal workpiece under continuous force application and varying working angles, aren values of 5.90 m/s^2^ at point 1 and 10.50 m/s^2^ at point 2 were recorded, together with noise levels of 90.0 and 91.3 dB(A), respectively. These findings suggest that the combination of tool power, task-related physical demands, and operational dynamics may have contributed to the increased vibration levels observed, representing the highest exposure intensity among the evaluated samples.

In the Automotive Maintenance/Welding sector, Welder 6 (1.70 m tall; 85 kg) performed finishing operations using a 7-inch Makita^®^ M0920 angle grinder equipped with a Norton flap disc. The task consisted of the surface finishing of semi-finished metal components, with varying tool application angles throughout the operation. Under these conditions, aren values of 5.20 m/s^2^ at point 1 and 9.10 m/s^2^ at point 2 were recorded, together with noise levels of 84.4 and 88.3 dB(A), respectively. Compared with the other samples, this condition yielded the lowest noise levels, suggesting that the use of a flap disc, composed of overlapping abrasive flaps, may be associated with lower noise emissions during grinding operations.

Overall, the results of the six samples demonstrated an asymmetric pattern of HAV exposure, regardless of the industrial sector, angle grinder power, or workers’ anthropometric characteristics. This pattern suggests that operational factors, such as the magnitude of the force applied during tool handling, the characteristics of the disc used, and the nature of the task performed, may substantially influence the observed exposure levels.

## DISCUSSION

Five measurements yielded aren values exceeding the tolerance limit of 5.0 m/s^2^ established by Annex 8 of NR 15 and NHO 10. These findings indicated high exposure levels in both hands of two welders and exclusively in the right hand of one welder. Under such conditions, the standard recommends the implementation of engineering controls if the pattern of angle grinder use remains unchanged [^7^].

Seven measurements produced aren values between 2.5 and 5.0 m/s^2^, corresponding to the range between the action value and the tolerance limit for HAV exposure. These values represent exposure conditions that already warrant the implementation of preventive measures, although they do not exceed the maximum permissible limit. Similar exposure ranges have also been reported in studies involving pole pruners, ride-on lawn mowers, chainsaws, and hedge trimmers [^11^,^14^], reinforcing the need for control measures in activities where these exposure levels are repeatedly encountered throughout the workday [^7^,^15^].

The comparison between Welder 4 and Welder 5, both using a 9-inch Bosch^®^ GWS 30-230 PB angle grinder equipped with a Norton BDA 640 grinding disc, revealed substantial differences in the recorded vibration levels. At the front handle, the aren value observed for Welder 5 (4.50 m/s^2^) was approximately 23.7% lower than that recorded for Welder 4 (5.90 m/s^2^). At the rear handle, the difference was even more pronounced, with aren values of 6.70 and 10.50 m/s^2^ respectively, which corresponds to an approximate reduction of 36.2%. These findings suggest that individual and operational factors, such as working posture, grip technique, and the magnitude of the applied force, may substantially influence the vibration transmitted to the upper limbs, even when the same tool is used to perform similar tasks [^14^,^16^-^18^].

In the current study, the differences observed in HAV and noise levels across the four assessments performed with 7-inch angle grinders demonstrated interindividual variability in exposure. Although the welders used similar tools and performed comparable tasks, the recorded exposure levels varied considerably between individuals. Similar findings have been reported by other authors and may reflect differences in tool handling as well as subtle variations in working conditions and equipment use [^14^,^18^].

According to the daily vibration exposure results obtained from the six angle grinder assessments (Chart 1), the highest vibration levels were consistently recorded at the rear handle, corresponding to the tool control hand. This asymmetric exposure pattern is consistent with previous findings for other vibrating tools [^11^,^14^], in which the rear handle, typically operated by the control hand, exhibits higher vibration levels than the front supporting handle [^2^,^15^,^18^,^19^].

In all samples, noise levels exceeded the action value established by current legislation [^7^]. However, unlike the pattern observed for HAV, noise exposure was similarly distributed between the two measurement points, with no marked lateral predominance.

## CONCLUSIONS

The present study characterized occupational exposure to HAV and noise during grinding operations across different industrial settings, highlighting the combined influence of technical, operational, and individual factors on the observed exposure levels. The findings demonstrated that, under all evaluated conditions, vibration and noise levels exceeded the action value and, in a subset of the samples, surpassed the tolerance limit established by Brazilian Law.

The analysis of exposure laterality showed that the highest vibration levels were consistently recorded at the rear handle, corresponding to the control hand operating the angle grinder. This pattern was observed for both 7-inch and 9-inch angle grinders and is consistent with previous studies demonstrating greater vibration transmission through the handles responsible for tool activation and control.

With regard to the regulatory framework, Annex 8 of NR 15 and NHO 10 neither require the simultaneous assessment of both hands when evaluating tools equipped with control and supporting handles nor establish objective criteria for defining significant differences between the aren values measured in each hand.

During some tasks, the workpieces remained unsecured while the angle grinder continued operating as workers visually inspected the surface finish without direct contact between the tool and the workpiece. Under these operating conditions, exposure to HAV and noise persisted even in the absence of active material removal. In addition, the high forces applied during grinding, together with abrupt tool deceleration upon contact with the workpiece, may have contributed to the increased vibration levels observed and, consequently, to higher daily worker exposure.

Based on these findings, future HAV exposure assessments should, whenever technically feasible, include measurements at both tool handles. This approach may provide a more representative estimate of exposure distribution, thereby supporting more informed decision-making in occupational health surveillance, occupational medicine, and safety engineering.

## Data Availability

The data supporting the findings of this study are available within the article.
